# Toll-Like Receptor 3 is Associated With the Risk of HCV Infection and HBV-Related Diseases

**DOI:** 10.1097/MD.0000000000002302

**Published:** 2016-05-27

**Authors:** Pei-Liang Geng, Li-Xue Song, Huaijie An, Jing-Yu Huang, Sheng Li, Xian-Tao Zeng

**Affiliations:** From the Center for Evidence-Based and Translational Medicine, Zhongnan Hospital of Wuhan University, Wuhan, China (PLG, JYH, SL, XTZ); Department of Pharmacology, General Hospital of Beijing Military Region, Beijing, China (LXS); Center of Basic Medical Sciences, Navy General Hospital of PLA Beijing, China (HA); and Department of Urology, Zhongnan Hospital of Wuhan University, Wuhan, China (JYH, SL).

## Abstract

There are inconsistent data on the association of risk of hepatitis virus infection and hepatitis virus-related diseases with the toll-like receptor 3 (*TLR3*) gene.

Several common polymorphism sites were targeted to assess the risk of HBV infection, HCV infection, and HBV-related diseases.

Meta-analysis combining data for 3547 cases and 2797 controls from 8 studies was performed in this study. Pooled ORs were calculated to measure the risk of hepatitis virus infection and hepatitis virus-related diseases. Fixed-effects pooled ORs were calculated using the Mantel-Haenszel method.

The *TLR3* gene was associated with a significantly increased risk of HBV-related diseases among 1355 patients and 1130 controls ([pooled OR, [95%CI]: 1.30, [1.15–1.48] for dominant; 1.77, [1.35–2.31] for recessive; 1.28 [1.16–1.41] for allele frequency). Subgroup analyses by a polymorphism site indicated an increased risk of HCV infection in relation to the TT/CT genotypes of rs3775291 (1.50 [1.11–2.01]), and a decreased risk ascribed to the T allele (0.20 [0.16–0.25]). We also noted an association between rs3775291 and significantly increased risk of HBV-related diseases (2.23 [1.55–3.21]). No significant inter-study heterogeneity or publication bias was detected in the analyses.

These data suggest a likely effect on the risk to infect HCV and develop HBV-related diseases for the *TLR3* gene. Large-scale studies with racially diverse populations are required to validate these findings.

## INTRODUCTION

Hepatitis B virus (HBV) and hepatitis C virus (HCV) are 2 ever-increasing health problems around the globe.^1^ More than 2,000,000,000 and 210,000,000 people worldwide have been affected by HBV and HCV, respectively.^2^ The 2 types of virus are considered to be major prevalent infectious agents and leading causes of severe liver diseases such as chronic hepatitis, liver cirrhosis, and hepatocellular carcinoma (HCC). A long list of conventional risk factors for HBV and HCV acquisition have previously been identified.^3^ In addition, genetic variations, typically single-nucleotide polymorphisms (SNP), in innate immunity receptors might be susceptibility factors for hepatitis virus infection.^4^

The innate immune system is a very important mechanism in defense against pathogenic intrusion. Delayed recognition and detection of the presence of infecting pathogens lead to organ dysfunction, inappropriate systemic responses, devastating tissue damage, life-threatening infections, and even death.^5^ Responses of the innate immune system are inducible and can be activated by pattern recognition receptors, which stimulate conserved host defense signaling pathways that regulate the expression of many immune response genes.^6^

Toll-like receptor 3 (TLR3) is a key pattern recognition receptor of the innate immune system. The *TLR3* gene triggers innate immune responses, enhances the production of cytokines essential for activation of innate immunity, and recognizes pathogen-associated molecular patterns expressed on infectious micro-organisms.

SNPs occur in >1% of the general population. They could induce amino acid conversions and thereby modify the promoter activities.^4,7^ Earlier research suggests significantly higher expression levels in chronic HCV infection patients with *TLR3* genotypes compared to their healthy counterparts.^7^ Recent research also demonstrates evidence of a higher infection rate in individuals who harbor SNPs of the *TLR3* gene that controls efficacy of innate immunity.^8^ Therefore, SNPs in *TLR3* may increase the risk of hepatitis virus infection and hepatitis virus-related diseases.

Multiple SNPs positioned in the TLR3 gene, especially those under investigation (rs1879026, rs3775296, rs3775291, rs5743305), have been targeted to assess the risk of HBV-, HCV infection, and related diseases.^9–16^ But the results are inconsistent. The objective of this study was to examine the associations between *TLR3* and risk of HBV infection, HCV infection, and HBV-related diseases by means of meta-analysis.

## METHODS

### Literature Search

We performed a systematic literature search in PubMed, Scopus, and Embase databases, without language restrictions. Search terms included toll-like receptor 3, *TLR3*, *CD283*, hepatitis, HBV infection, HCV infection, polymorphism, polymorphisms, variants, and variations. These words were used in combination or in isolation. Two of the authors singled out all publications addressing the association of interest and examined their reference lists. They also checked the references cited in related review articles to obtain additional data. The study was approved by the institutional review board. All patients provided written informed consent.

### Selection of Studies

Selection of studies eligible for inclusion was based on the following criteria defined before the commencement of publication search. Whenever 2 or more studies were overlapped in terms of subjects, we retained the most complete and informative study with a larger sample size.

### Inclusion Criteria

- Included patients with HBV-infection, HCV-infection or HBV-related disease, and non-infected or healthy controls;
- Examined the association of SNPs in the *TLR3* gene with risk of HBV infection, HCV infection, or HBV-related disease;
- Clearly reported the genotype frequencies of each polymorphism.
- Genotype distribution in controls must be in accordance with Hardy–Weinberg equilibrium (HWE).

### Exclusion Criteria

- Editorials, reviews, comments, abstracts with insufficient data, and conference proceedings.
- Full-length articles with inaccessible genetic data.
- Only patients were included.
- Studies that deviated from HWE.

### Data Extraction

Data for each study were separately extracted by 2 authors using a standard form. Disagreements were resolved by consensus including a third author. Information on the following characteristics was recorded: first author, year of publication, polymorphisms studied, infection type, disease type, number of genotypes, total cases and controls, location in which the study was conducted, ethnicity of study subjects, proportion of males, and study setting.

### Statistical Analysis

Pooled odds ratios (ORs) were estimated to assess the relationship between *TLR3* SNPs and risk of HBV-infection, HCV-infection, and HBV-related diseases. The ORs were calculated by comparing the mutated homozygote/heterozygote genotypes (22/12) with the wide-type genotype (11), then 22 with 12/11 in a recessive model, and 2 with 1 in an allele frequency model. Heterogeneity assumption was measured by the chi-square-based *Q* test,^17^ with *P* value <0.05 considered significant. The summary ORs with the corresponding 95% confidence intervals (95%CI) were calculated with the fixed-effects or the random-effects model. Random-effects summary ORs were calculated using the DerSimonian–Laird method when significant heterogeneity presented.^18^ The Mantel–Haenszel method was utilized to calculate fixed-effects summary ORs in case of the absence of heterogeneity.^19^ HWE was checked by the chi-square test in the controls. Publication bias was determined by funnel plot and Egger's test,^20^ a type of linear regression methodology widely used to measure funnel plot asymmetry. Sensitivity analyses were performed to examine the validity of meta-analysis results. Statistical analyses were done by the STATA software (package v.12.0). *P* < 0.05 was considered statistically significant.

## RESULTS

### Studies Included in the Meta-Analysis

**FIGURE 1:**
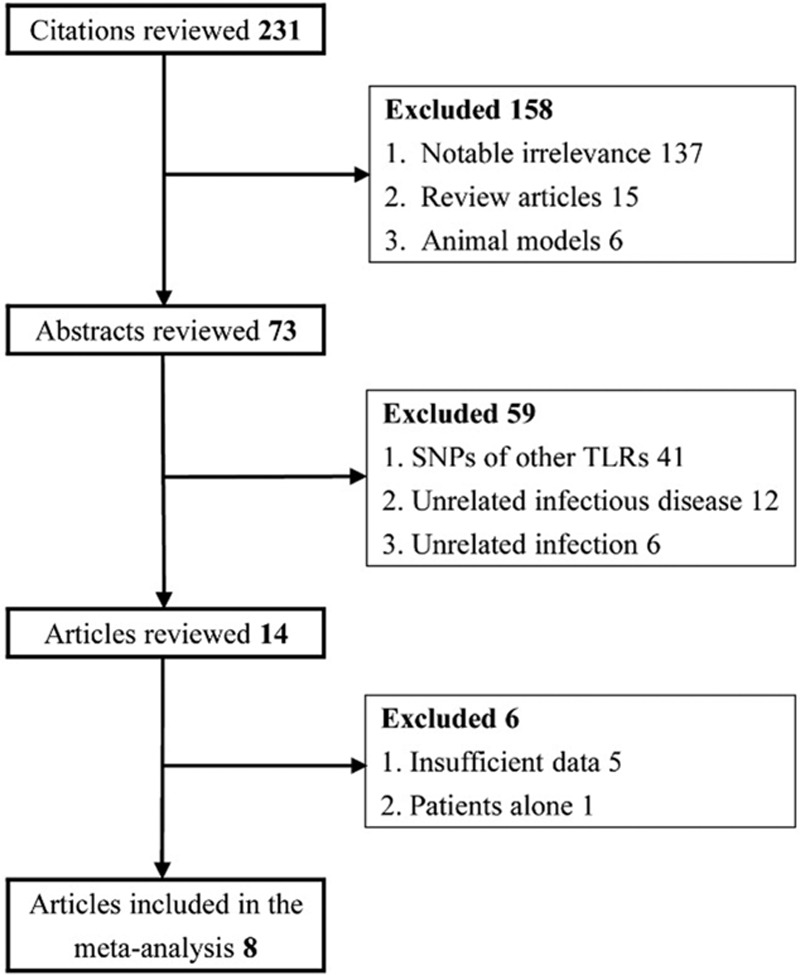
The low diagram of included/excluded studies.

A total of 231 citations were identified through databases. Around 158 citations were excluded after title review. We then reviewed 73 abstracts. Among these, 59 articles not associated with *TLR3* SNPs, hepatitis virus infection or hepatitis virus-related diseases were excluded. Of the14 articles whose eligibility could not be determined by title and abstract review, 6 were discarded after full-text review. The specific reasons for study exclusion and inclusion are described in Figure 1. Thus, 8 articles were included in the final analysis,^9–16^ providing 3547 cases and 2797 controls. Asian subjects were used in most studies (62.5%, n = 5). In addition, 2 studies employed North Americans and 1 enrolled South Americans. Regarding the setting of study, 5 included healthy individuals, 2 used individuals with previous HBV clearance, and 1 included non-HCV-infected liver recipients (Table 1).

**TABLE 1:**
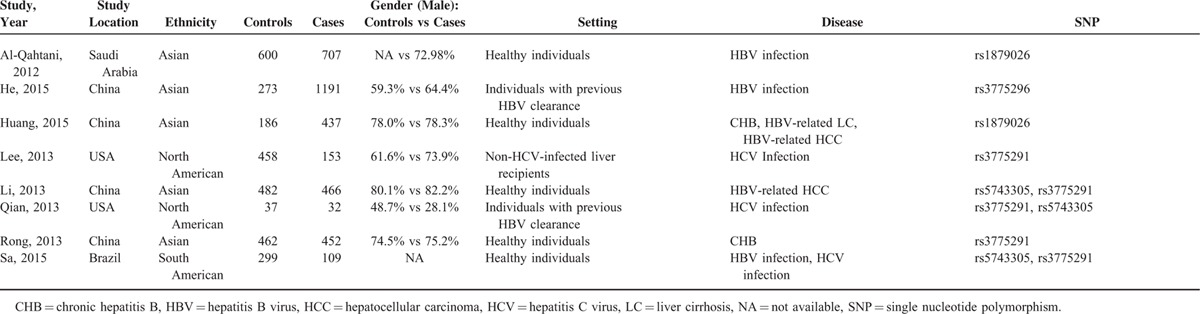
Primary Characteristics of Studies Incorporated in Meta-Analysis

### Association of *TLR3* Polymorphisms With HBV, HCV Infection

**FIGURE 2:**
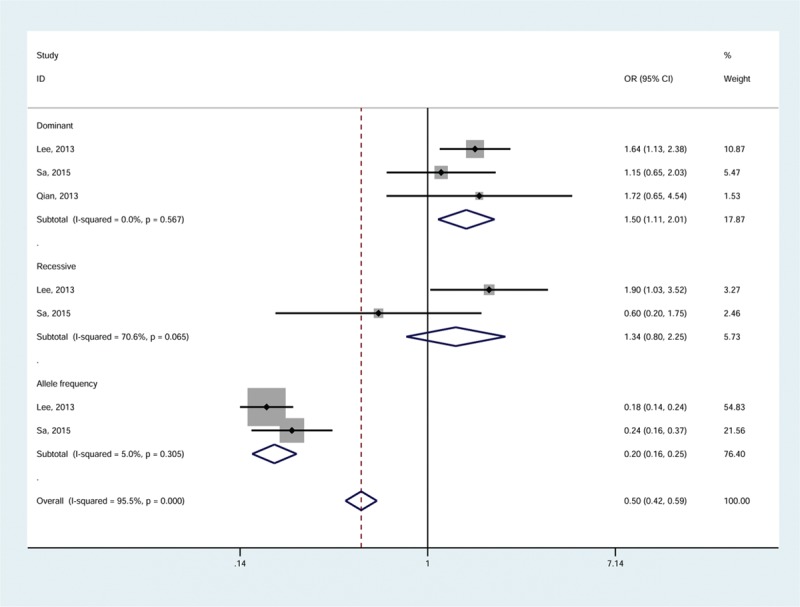
Meta-analysis for association between rs3775291 and the risk of HCV infection by the fixed-effects model. HCV = hepatitis C virus.

There was no association observed between the *TLR3* polymorphisms and HBV infection (pooled OR [95%CI]: 1.30 [1.15–1.48} for dominant; 1.77 [1.35–2.31] for recessive; 1.28 (1.16–1.41) for allele frequency, Table 2). Also, no significant association was revealed in the analysis of HCV infection (1.26 (0.99–1.61) for dominant; 1.22 (0.77–1.92) for recessive; 1.19 (0.97–1.45) for allele frequency, Table 2). Subgroup analyses by polymorphism site indicated a significant increase in the risk of HCV infection associated with the TT/CT genotypes at rs3775291 [1.50, (1.11–2.01)]. Intriguingly, a significantly decreased risk was associated with the rs3775291-T allele [0.20, (0.16–0.25)]. The associations are illustrated in Figure 2.

**TABLE 2:**
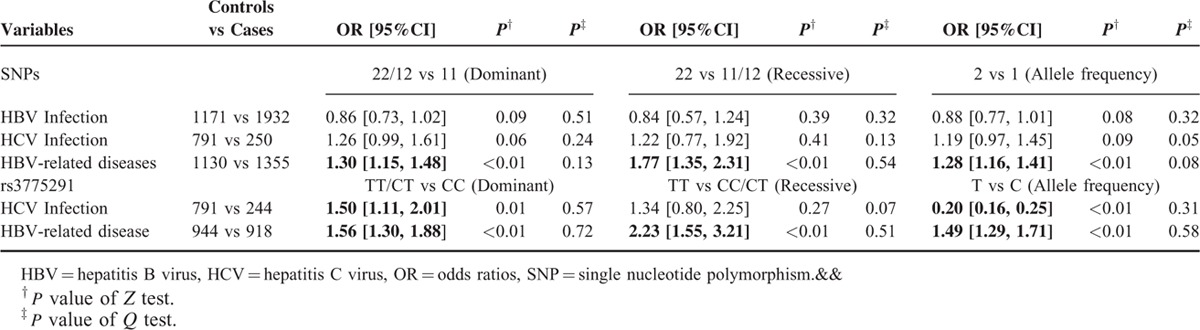
Impact of TLR3 on Risk of Hepatitis Virus Infection and Hepatitis Virus-Related Diseases Using the Fixed-Effects Model

### Association of *TLR3* Polymorphisms With HBV-Related Diseases

**FIGURE 3:**
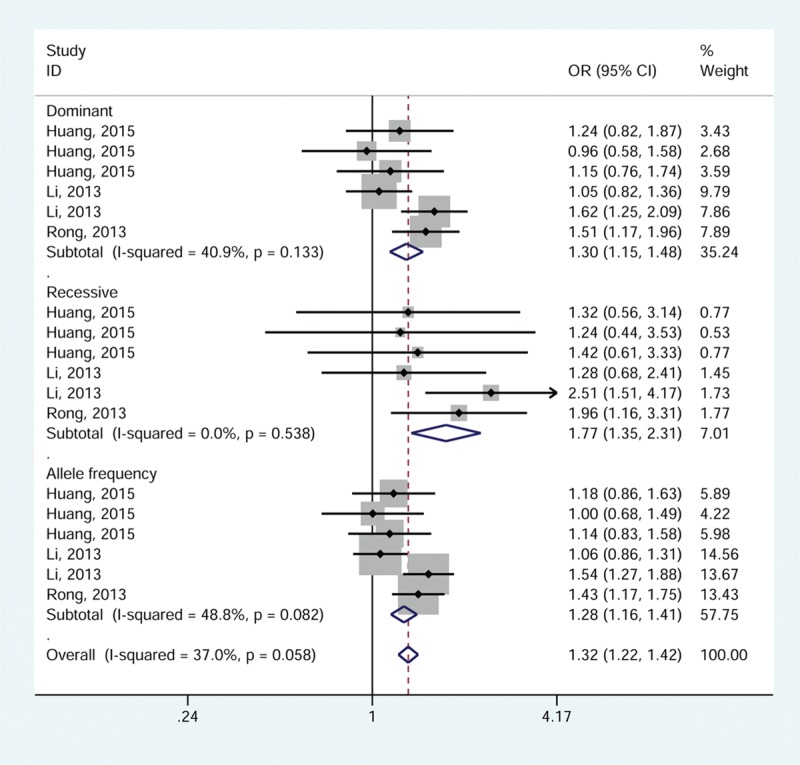
Meta-analysis for association between *TLR3* polymorphisms and the risk of HBV-related diseases by the fixed-effects model.

Using the Mantel–Haenszel method, the polymorphisms in the *TLR3* gene were associated with a significantly increased risk of HBV-related diseases. The pooled OR was greatest in the recessive model (1.77 [1.35–2.31]), and slightly lower in dominant model (1.30 [1.15–1.48]) and allele frequency model (1.28 [1.16–1.41]). The forest plot is shown in Figure 3.

In the subgroup analysis according to polymorphism site, there was a 1.56- to 2.23-fold increased risk in relation to rs3775291 [1.56 (1.30–1.88) for dominant; 2.23, (1.55–3.21) for recessive; 1.49 (1.29–1.71) for allele frequency, Table 2].

### Sensitivity Analysis

Sensitivity analyses by meta-analysis in the absence of 1 single study were conducted to examine the stability of the combined risk estimates. Statistical significance of the summary ORs was not substantially modified (data not shown). Therefore, our results are stable.

### Publication Bias

Funnel plots were created by the standard error plotting against the OR for each study. No evidence of asymmetry was indicated in the funnel plots (*P* > 0.05). Egger's linear regression test provided data that supported the absence of publication bias in the meta-analysis (*P* > 0.05).

## DISCUSSION

As far as we are aware, this is the first study by use of meta-analysis to examine the relationship between polymorphisms in the *TLR3* gene and risk of hepatitis virus infection and hepatitis virus-related diseases. Overall, we found a significantly increased risk of HBV-related diseases in relation to the polymorphisms tested. The association remained highly significant when the data were restricted to rs3775291. A strong association was also observed in the analyses of rs3775291 and HCV infection. But it is interesting that the TT/CT genotypes were associated with increased risk of HCV infection and the T allele protected against the virus infection. There was no evidence of high possibility of publication bias or significant interstudy heterogeneity.

The strong association observed in our analysis is consistent with the biological properties of *TLR3*. TLR3 is a known pattern-recognizing receptor with an important part in immune signaling and innate immune recognition of intruding microbes, stimulating adequate immune responses. A growing body of literature has suggested that SNPs in the *TLR* genes attenuate the ability of some individuals to respond appropriately to TLR ligands, and this attenuation may lead to increased occurrence of infections and infectious diseases.^21^ Results of a recent study support the previous finding. Ranjith-Kumar et al suggested that several polymorphisms that alter TLR3 amino acids initiate resultant changes in the protein and that they might downregulate the gene expression and lower the activities of TLR3 required for proper signaling.^22^ Reduced activity of TLR3 results in failure to recognize invading microorganisms and insufficient immune responses, thus increasing the likelihood of infections and infectious diseases. All data point to the importance of *TLR3* SNPs in the development of infections and infection-induced diseases.

The association between rs3775291 polymorphism in the coding region of the *TLR3* gene and risk of HCV infection and HBV-related diseases is also biologically plausible. This extensively studied polymorphism results in amino acid substitution. Mutations at this site may destroy the structure and impair the function of the protein due to the highest purifying selection pressure related to the C allele.^22^ The amino acid leucine at rs3775291 is adjacent to an asparagine whose glycan moiety binds double-stranded RNA (dsRNA),^23^ a replication intermediate of many viruses. As mutations in the neighboring region (Asp413) result in significantly reduced signaling activity of TLR-3, rs3775291 may prevent glycan moiety of Asp413 from binding to dsRNA or influence its glycosylation, which seems to be able to explain the reduced signaling activity induced by rs3775291.^24,25^ Again, reduction in signaling activity of TLR-3 causes higher incidence of infections and related diseases. Therefore, it is likely that the rs3775291 polymorphism is associated with the risk of HCV infection and HBV-related diseases. Our findings are also in concordance with a recent observation that HCV per se may induce downregulation of TLR3 expression on innate immune cells.^29^

Several investigations have examined the association of *TLR3* polymorphisms with hepatitis virus acquisition and hepatitis virus-related diseases. Al-Qahtani et al included 707 patients infected with HBV and 600 uninfected controls of Arabian descent, demonstrating a significant effect of genetic variations in the *TLR3* gene on the outcome of HBV infection.^9^ The positive finding was confirmed in a replication study among more than 1400 subjects of Han Chinese descent.^10^ In a most recent analysis, Huang et al likewise suggested a significant association between *TLR3* SNPs and HBV-induced diseases.^11^ Unlike the aforementioned analyses, Sa et al showed the absence of an association between *TLR3* polymorphisms and susceptibility to HBV and HCV infection in 109 cases and 299 healthy controls of South American descent.^16^ Different sample sizes are a possible explanation for the conflicting results. Another important reason may relate to ethnicity variance. According to Abdel-Hady et al, HBV infection is most prevalent in Asia, Africa, Southern Europe, and Latin America.^26^ With respect to HCV-related infection and related diseases, the prevalence is relatively lower in economically developed countries including Africa, the West Pacific, Eastern Mediterranean, and South-East Asia, than in less developed countries including northern and western Europe, North America, and Australia (1–2% vs 5–10%).^27,28^ Based on these data, we infer that there may be ethnic differences in the influence of *TLR3* polymorphisms.

Some limitations should be noticed in interpreting the results. First, since most of the polymorphisms being investigated were evaluated in a low number of studies, we were not able to detect their effects individually, possibly resulting in overgeneralized results. Second, we observed significant effects when all published data were combined. However, the impact of *TLR3* polymorphisms may be ethnicity-specific. It is possible that the genetic polymorphisms with a significant role in one ethnic group may not have equal impact on another. Hence analyses restricted to certain specific ethnic groups are necessary. Third, despite the potential confounding effect conferred by age, gender, lifestyle, and environmental risk factors, we were not allowed to evaluate these effects in our study as a result of data inadequacy.

In summary, polymorphisms of the *TLR3* gene may be potential risk factors for HCV infection and HBV-related diseases. Further analyses incorporating a larger number of samples and ethnically different populations are warranted to determine the association between *TLR3* and risk of hepatitis virus infection and hepatitis virus-related diseases.
